# Local molecular analysis of indeterminate thyroid nodules

**DOI:** 10.1186/s40463-015-0106-2

**Published:** 2015-12-01

**Authors:** Mandeep S. Gill, Smriti Nayan, Linda Kocovski, Jean-Claude Cutz, Stuart D. Archibald, Bernard S. Jackson, James E. M. Young, Michael K. Gupta

**Affiliations:** Division of Otolaryngology and Head and Neck Surgery, Department of Surgery, McMaster University, Hamilton, ON Canada; Department of Pathology, McMaster University, Hamilton, ON Canada

## Abstract

**Background:**

Thyroid nodules are common but only a minority are malignant. Molecular testing can assist in helping determine whether indeterminate nodules are suspicious for malignancy or benign. The objective of the study was to determine if the analysis of mutations (BRAF, NRAS, KRAS and HRAS) using readily available molecular techniques can help better classify indeterminate thyroid nodules.

**Methods:**

A retrospective cohort of consecutive patients undergoing diagnostic thyroid surgery were analyzed for the presence or absence of specific mutations known to be associated with thyroid malignancy in FNA samples. Markers chosen were BRAF, NRAS, KRAS and HRAS. All were locally available and currently in use at our centre for other clinical indications. Results from the molecular analysis were then compared to the histopathology from thyroidectomy specimens to determine the sensitivity and specificity of these molecular techniques to classify indeterminate thyroid nodules.

**Results:**

Sixty consecutive patients with indeterminate FNAs were recruited. Twenty-three patients had malignant tumors while 37 specimens were benign. Multiple different mutations were identified in the FNA samples. Overall 18 cases had a positive mutation (10 malignant and 8 benign). The sensitivity of BRAF, HRAS, KRAS, and NRAS was 8.7, 8.7, 8.7, and 17.4 respectively while the specificity was100, 83.7, 100 and 94.6.

**Conclusion:**

While molecular analysis remains promising, it requires further refinement. Several markers showed promise as good "rule-in" tests.

## Background

The overall incidence of thyroid nodules in the general public is estimated to be between 20-75 % [1]. A minority of these nodules are malignant. The standard work-up for thyroid nodules includes a thyroid stimulating hormone level, an ultrasound, and a fine needle aspiration biopsy (FNAB). After completion of this standard work-up, about 20 % of these remain indeterminate [2]. The proportion of malignancy in this group is around 20 % [3]. Further classification of these nodules may rely on repeat FNAB, serial ultrasound follow-up, or diagnostic thyroid surgery. These options are imperfect as repeat FNAB is often unsuccessful at further classifying these nodules, serial ultrasounds assume the small risk of surveilling cancer, and diagnostic thyroid surgery is invasive, has non-trivial risks and is often done for benign disease. In addition, the uncertainty surrounding indeterminate thyroid lesions can be a great consternation to patients [4].

Preoperative molecular analysis of the contents of FNA has been an emerging field in thyroid research as it tries to better classify thyroid nodules as benign or malignant. Multiple mutations have been identified in thyroid cancer and studied for their predictive ability in FNA specimens. These include b-raf murine sarcoma viral oncogene homolog B (BRAF), neuroblastoma RAS viral oncogene homolog (NRAS), Kirsten rat sarcoma viral oncogene homolog (KRAS), peroxisome, proliferator-activated receptor γ (PPARG), and the thyroid stimulating hormone receptor gene (TSHR) among others [5–7].

Building on this previous work, novel molecular tests such as, the Afirma Gene Expression Classifier and ThyroSeq v2 have been created [6, 8]. These molecular tests analyze specimen from FNAB simultaneously testing for the presence of multiple mutations.

These molecular techniques are enormously promising. Implementing highly accurate molecular assessments of indeterminate thyroid lesions could lift patients and physicians out of the miasma that is the indeterminate thyroid nodule. Anxiety surrounding the diagnosis could be eliminated, invasive surgery could be avoided and serial ultrasounds could be halted.

Unfortunately, in their current form the most sophisticated and accurate molecular tests such as Afirma and Thyroseq v2 are costly and, unavailable in Canada. Consequently, our group decided to study the use molecular markers that have been shown to be predictive of malignancy in indeterminate samples and are already in use at our institution. The hope of this study was that by creating a pragmatic assay of molecular markers we could refine our diagnosis of indeterminate thyroid lesions.

The primary outcome of this pilot study was to assess feasibility and if molecular testing could be conducted based on the current infrastructure at our center. The secondary outcomes were the sensitivity and specificity of the tests to predict thyroid cancer and to determine the proportion of cancer in indeterminate samples undergoing recruitment.

## Methods

A retrospective pilot study was conducted by reviewing patient charts from the Head and Neck Clinics of four Otolaryngology – Head & Neck Surgeons at St. Josephs Healthcare Hamilton. Hamilton Integrated Research Ethics Board (HIREB #12-3667) approval was obtained prior to beginning the study. Records spanning the period of August 2007 to July 2012 were examined for all patients who had a thyroid FNAB with indeterminate cytology. Indeterminate cytology comprises 3 recognized Bethesda diagnostic categories: atypia or follicular lesion of undetermined significance (AUS/FLUS), follicular neoplasm or suspicious for follicular neoplasm (FN/SFN). The inclusion criteria were patients who had thyroid FNA with indeterminate cytology, a final surgical excision diagnosis, were older than 18 years of age and had moderate to good cellularity on FNA. Exclusion criteria included patients with a family history of thyroid cancer, history of radiation exposure and those who did not have a final surgical excision diagnosis. A minimum sample size of 220 was calculated based upon published values from previous studies of molecular testing in thyroid cancer. A sample size of 60 was used for this feasibility study. Using values of a sensitivity of 60 %, a specificity of 92 %, and a prevalence of carcinoma in indeterminate FNA results as 20 %, and a desired precision of 15 %, the sample size was calculated to be 60. Once patients were identified who satisfied the inclusion and exclusion criteria their cytology slides and cell blocks were reviewed for cellularity and sample adequacy i.e. thyroid follicle cells present, not just blood, colloid and/or inflammatory cells. A tissue section was cut from each cell block, stained with H&E, and examined to confirm cellularity. Two independent assessors (pathologist and pathology resident) who were blinded to both the FNA and final excisional diagnoses examined each case. Those cases that had adequate cellularity (defined as presence of thyroid follicle cells) were included in the study.

### Molecular analysis

A 7 μm tissue curl was cut from each cell block and treated with TrimGen Wax*Free™* DNA extraction kit for formalin-fixed paraffin embedded tissue. Molecular analysis was conducted for oncogenes using polymerase chain reaction (PCR) and pyrosequencing (Qiagen): BRAF codon V600 mutations: V600E/K/D/R, KRAS codons 12/13 mutations: G12A/D/R/C/S/V, G13D, NRAS codon 61 mutations: Q61R/K/L/H/P/E/Q and HRAS codon 61 mutations: Q61R/K/L/H/P. These were then compared to the result of the final surgical specimen [5–7, 9–12]. These were used as they were currently available at our institution and being used clinically for other purposes.

## Results

Sixty patients were identified in the retrospective chart review who met the inclusion and exclusion criteria. The FNA samples were sent for molecular analysis for mutations in BRAF, KRAS, NRAS and HRAS. It was feasible to run all testing locally. Overall, of the 60 patients who had indeterminate results on FNA, 37 (62 %) were benign lesions while 23 (38 %) had malignant lesions when the final surgical specimens were analyzed. The most common benign entity was follicular adenoma while the most common malignant entity was papillary carcinoma, classical variant (Table 1). There were 2 patients who had mutations in BRAF, 8 patients who had mutations in HRAS, 2 patients who had mutations in KRAS and 6 patients who had mutations in NRAS. Overall there were 18 mutations that were identified in the 60 patients of this study. Of these 10 ended up being malignant and 8 ended up having benign pathology. BRAF had a specificity of 8.7 %, a sensitivity of 100 %, a positive predictive value of 100 % and a negative predictive value of 63.8 %. HRAS had a specificity of 8.7 %, a sensitivity of 83.7 %, a positive predictive value of 25 % and a negative predictive value of 59.6 %. KRAS had a specificity of 8.7 %, a sensitivity of 100 %, a positive predictive value of 100 % and a negative predictive value of 63.8 %. NRAS had a specificity of 17.4 %, a sensitivity of 94.6 %, a positive predictive value of 66.7 % and a negative predictive value of 64.8 %. Overall the combined specificity was 43.5 %, the sensitivity was 78.4 %, a positive predictive value of 55.5 % and a negative predictive value of 69 % (Table 2).

**Table 1:** Histological analysis of final specimens

Benign		Malignant	
Nodular/adenomatous hyperplasia	7 (12 %)	Papillary carcinoma, classical variant	9 (15 %)
Adenomatous colloid nodule	8 (13 %)	Papillary carcinoma, follicular variant	7 (12 %)
Hurthle cell adenoma	1 (2 %)	Papillary carcinoma, microcarcinoma variant	5 (8 %)
Follicular adenoma	17 (28 %)	Papillary carcinoma, oncocytic variant	1 (2 %)
Atypical follicular adenoma	4 (7 %)	Poorly differentiated follicular carcinoma	1 (2 %)
Total	37 (62 %)	Total	23 (38 %)

**Table 2:** Sensitivity, specificity, positive and negative predictive value of each gene

Molecular Mutation	Number of Positive Cases	Malignant	Benign	Sp	Sn	PPV	NPV
BRAF	2	2	0	8.7	100	100	63.8
HRAS	8	2	6	8.7	83.7	25	59.6
KRAS	2	2	0	8.7	100	100	63.8
NRAS	6	4	2	17.4	94.6	66.7	64.8
Overall	18	10	8	43.5	78.4	55.5	69

## Discussion

The primary outcome was to determine if it was feasible to run molecular testing at our center with current resources. It was determined that testing could be done locally at our center and results were obtained in a timely fashion. There have been various studies illustrating the effectiveness of Afirma and ThyroSeq v2 to determine whether indeterminate lesions are benign or malignancy. While both are effective testing modalities, their high costs make it difficult to institute on a daily basis and they are both not currently available in Canada. The Afirma testing modality looks at the mRNA expression of 167 genes and the ThyroSeq v2 looks at 13 genes and for 42 types of gene fusions that occur in thyroid cancer. This study was designed to be much simpler and as such looked at only looked at 4 molecular genes. Afirma has a published sensitivity of 92 %, a specificity of 52 % and a negative predictive value of greater than 90 %. The ThyroSeq v2 has shown a sensitivity of 90 %, a specificity of 93 %, a positive predictive value of 83 % and a negative predictive value of 96 % [6, 8]. Overall the combined specificity was 43.5 %, the sensitivity was 78.4 %, the positive predictive value was 55.5 % and the negative predictive value was 69 % for the four genes that were looked at in this study. There was a great deal of variability in the markers themselves in this study with sensitivity varying from 8.7 % to 17.4 % and specificity varying from 83.7 % to 100 %.

Although this study did not approach the values for sensitivity and specificity seen in studies on Afirma and ThyroSeg v2, a few of the tests were useful if the test was positive. This suggests that perhaps a relatively simpler set of genetic tests could be created as a rule-in test.

This study was a feasibility study. Certainly any results from this study require further refinement. As it was primarily a feasibility study, the molecular analysis that was conducted was done with genes that were already looked at our center for other clinical indications and not preselected for their effectiveness at determining thyroid cancer. There was no other reason as to why these collective genes were chosen besides being convenient and cost saving. Obviously, the construction of a more complete molecular test requires inclusion of mutation testing beyond what is readily available at our institution. It seems that that combination of mutations could hold promise in helping determine which patients have benign nodules and which ones are suspicious for malignancy. It has the ability to provide additional information to patients when they are making decisions on how to proceed with an indeterminate thyroid nodule. Moreover, it has the potential to reduce the number of diagnostic hemithyroidectomies which are invasive, costly, anxiety provoking, and like all surgical procedures; they have associated risks of complications. Thus, even though there were limitations of this study it still can be promising in providing patients with additional information. It is unclear how effective this set of four genes will be in terms of trying to delineate which thyroid nodules are benign and which ones are malignant.

One of the secondary outcome was the proportion of malignancy in the sample of 60 indeterminate FNAs. The proportion of malignancy in any sample depends on many site specific factors. In this study, 38 % of these lesions were malignant. This prevalence compares favourably with the literature as values of roughly 15-30 % of indeterminate lesions are malignant are reported in the literature [6]. The slightly higher rate of malignancy raises the question of external validity of the findings. However, concerns of this nature can be allayed by the fact that the prevalence was only slightly different and the fact that the sensitivity and specificity of a test is independent of the prevalence in a population.

There are several limitations to this study. It is important to note that a pragmatic cohort was taken in this pilot study. We do recognize the limitations of this and a more generalizable cohort would be more applicable in determining the sensitivity and specificity of such tests in a clinical setting. Being a pilot study we limited the cohort to patients who underwent surgery. Moreover, economic analysis and evaluation was not completed in this pilot study as it was deemed not reasonable as we are in the process of determining the efficacy and effectiveness of such analysis. The small sample size is a limitation of the study and the true sensitivity and specificity of these combinations of markers will require a larger sample size. It is clear that larger samples sizes are needed to determine if these clinical markers will have an impact on the management of patients with indeterminate thyroid nodules. These clinical markers can aid in providing patients more information when making decisions but it is unclear the impact these four markers will have in changing the recommendations of management.

The study illustrates the promise in potentially finding a more convenient way to complete molecular analysis on thyroid nodules with local resources. Moreover, with the expansions in genetic thyroid research molecular testing has the potential to change clinical practice for patients suffering from thyroid nodules. The molecular analysis of FNA specimens remains in its infancy. It is likely though, that over the next several years more molecular tests will become available and will become integrated into clinical practice. Which tests are chosen and how they are integrated into practice will certainly be among the most important decisions facing thyroid surgery in the coming years.

## Conclusions

Overall there may be some benefit in the use of locally available molecular analysis and testing in determining malignancy in thyroid nodules. Despite a limited sample size, molecular testing remains promising even with this limited group of four genes.
